# Clinical characteristics and predictors of mortality associated with COVID-19 in elderly patients from a long-term care facility

**DOI:** 10.1038/s41598-020-77641-7

**Published:** 2020-11-30

**Authors:** Enrico Maria Trecarichi, Maria Mazzitelli, Francesca Serapide, Maria Chiara Pelle, Bruno Tassone, Eugenio Arrighi, Graziella Perri, Paolo Fusco, Vincenzo Scaglione, Chiara Davoli, Rosaria Lionello, Valentina La Gamba, Giuseppina Marrazzo, Maria Teresa Busceti, Amerigo Giudice, Marco Ricchio, Anna Cancelliere, Elena Lio, Giada Procopio, Francesco Saverio Costanzo, Daniela Patrizia Foti, Giovanni Matera, Carlo Torti, IDTM UMG COVID-19 Group, Domenico Laganà, Maria Petullà, Bernardo Bertucci, Angela Quirino, Giorgio Settimo Barreca, Aida Giancotti, Luigia Gallo, Angelo Lamberti, Maria Carla Liberto, Nadia Marascio, Adele Emanuela De Francesco

**Affiliations:** 1 Infectious and Tropical Disease Unit, Department of Medical and Surgical Sciences, “Magna Graecia” University of Catanzaro, Viale Europa, 88100 Catanzaro, Italy; 2 Respiratory Unit, “Mater Domini” Teaching Hospital, Catanzaro, Italy; 3 Department of Health Sciences, “Magna Graecia” University of Catanzaro, Catanzaro, Italy; 4 Department of Experimental and Clinical Medicine, “Magna Graecia” University of Catanzaro, Catanzaro, Italy; 5 Center of Interdepartmental Services (CIS), “Magna Graecia” University of Catanzaro, Catanzaro, Italy; 6 Clinical Pathology Unit, Department of Health Sciences, “Magna Graecia” University of Catanzaro, Catanzaro, Italy; 7 Department of Health Sciences, Institute of Microbiology, “Magna Graecia” University of Catanzaro, Catanzaro, Italy; 8 Radiology Operating Unit, Department of Clinical and Experimental Medicine, “Mater Domini” Teaching Hospital, Catanzaro, Italy; 9 Microbiology Unit, “Mater Domini” Teaching Hospital, Catanzaro, Italy; 10 Pharmacy Unit Department, “Mater Domini” Teaching Hospital, Catanzaro, Italy

**Keywords:** Infectious diseases, Epidemiology

## Abstract

Since December 2019, coronavirus disease 2019 (COVID-19) pandemic has spread from China all over the world and many COVID-19 outbreaks have been reported in long-term care facilities (LCTF). However, data on clinical characteristics and prognostic factors in such settings are scarce. We conducted a retrospective, observational cohort study to assess clinical characteristics and baseline predictors of mortality of COVID-19 patients hospitalized after an outbreak of SARS-CoV-2 infection in a LTCF. A total of 50 patients were included. Mean age was 80 years (SD, 12 years), and 24/50 (57.1%) patients were males. The overall in-hospital mortality rate was 32%. At Cox regression analysis, significant predictors of in-hospital mortality were: hypernatremia (HR 9.12), lymphocyte count < 1000 cells/µL (HR 7.45), cardiovascular diseases other than hypertension (HR 6.41), and higher levels of serum interleukin-6 (IL-6, pg/mL) (HR 1.005). Our study shows a high in-hospital mortality rate in a cohort of elderly patients with COVID-19 and hypernatremia, lymphopenia, CVD other than hypertension, and higher IL-6 serum levels were identified as independent predictors of in-hospital mortality. Given the small population size as major limitation of our study, further investigations are necessary to better understand and confirm our findings in elderly patients.

## Introduction

Since SARS-CoV-2 pandemic has spread from China all over the world, more than 3,700,000 people have been infected, with almost 260,000 of reported deaths until May 8th, 2020^1^. The spectrum of clinical manifestations of SARS-CoV-2 associated disease (COVID-19), varies from the absence of symptoms to severe disease, eventually leading to death. COVID-19 has been reported to be severe and critical in 14% and 5% patients, respectively^2^. Since the first clinical reports on COVID-19, older age and coexisting comorbidities, in particular cardiovascular or cerebrovascular diseases, have been highlighted as risk factors associated with an adverse outcome of COVID-19^3–6^. These clinical characteristics are common among residents in Long-Term Care Facility (LTCFs). Noteworthy, these institutions can act as incubators of infection and elderly who live in LTCF are at higher risk of SARS-CoV-2 infection^7^. Several COVID-19 outbreaks have been reported in different countries, including Italy, with high mortality rates, reaching 49–64% in some countries^8^. Although there are studies describing characteristics of such patients and dynamics of transmission in this setting^9^, there is a lack of analyses investigating predictors of mortality in geriatric patients. These results are important to guide clinical management. Herein, we report clinical characteristics, outcome (in-hospital mortality), and prognostic factors in a cohort of 50 patients as part of an outbreak of SARS-CoV-2 infection in an Italian LTCF.

## Results

In the present study, clinical characteristics and outcome of a cohort of 50 patients diagnosed with COVID-19 who were transferred from a LTCF to the Infectious and Tropical Disease Unit of "Mater Domini" Teaching Hospital, Catanzaro, Italy are described. Out of 50 patients, 49 were diagnosed with COVID-19 by positive SARS-CoV-2 molecular test conducted on nasopharyngeal swab; the remaining patient was diagnosed on the basis of epidemiological link and clinical/radiological findings. Mean age was 80 years (SD, 12 years), and 24/50 (57.1%) patients were males.

In Table 1, clinical and demographic characteristics and outcome of the study patients are reported. The majority of patients suffered from cardiovascular diseases (CVD, 82%) and/or neurological diseases (52%); 52% of patients presented more than two comorbidities.

**Table 1:** Clinical and demographic characteristics and outcome of patients with COVID-19 coming from a LTCF.

Variables	Total (n = 50)
**Demographic information**	
Male sex	28 (57.1)
Age (years, mean ± SD)	80 ± 12
**Comorbidities and previous drug therapies**	
Cancer	9 (18)
Cardiovascular diseases (CVD)	41 (82)
CVD other than hypertension	19 (38)
Psychiatric disorders	15 (30)
Neurologic diseases	26 (52)
Obesity	5 (10)
Diabetes mellitus	11 (22)
COPD	9 (18)
Chronic renal failure	20 (40)
> 2 coexisting comorbidities	26 (52)
Bedridden status	30 (60)
Polipharmacy	36 (72)
Number of medications (mean ± SD)	6 ± 3
ACE-I/ARB therapy	16 (32)
Absence of typical COVID19-associated symptoms at initial presentation^a^	8 (16)
Moderate COVID-19 disease (NIH criteria)	24 (48)
Severe COVID-19 disease (NIH criteria)	26 (52)
**Outcome scores and mortality**	
*Brixia* score (mean ± SD)	7 ± 3
CALL score (mean ± SD)	11 ± 2
In-hospital mortality	16 (32)

*CVD* cardiovascular diseases, *ACE-I* angiotensin-converting-enzyme inhibitors, *ARBs* angiotensin-receptor blockers, *NIH* National Institutes of Health.

^a^Typical COVID-19-associated clinical symptoms at initial presentation included: fever, cough, dyspnea.

The overall in-hospital mortality rate was 32% (16/50).

A total of 38/50 (76%) patients received combination therapy with hydroxychloroquine plus azithromycin according to the study protocol by Gautret et al.^10^, with frequent electrocardiographic monitoring and without cardiac complications. Moreover, all the patients received anticoagulant therapy with enoxaparin (44 patients) or fondaparinux (6 patients); in 26/50 patients (52%) anticoagulants were administered at therapeutic dosage, whereas in the remaining 24 (48%) at prophylactic dosage. Finally, on the basis of clinical judgment (e.g., severe acute respiratory failure), in 25/50 (50%) patients corticosteroid therapy with intravenous methylprednisolone was administered. Similarly, on the basis of clinical judgment (e.g., severe acute respiratory failure) and high IL-6 levels, two patients were treated with tocilizumab single dose (162 mg) administered subcutaneously, as described elsewhere^11^.

### Risk factors for in-hospital mortality

Analyses of risk factors for in-hospital mortality have been conducted in 48/50 patients; two patients were excluded because they died few hours from hospital admission and thereof complete dataset was lacking. At univariate Cox regression analysis (described in Table 2), variables associated with in-hospital mortality were: CVD other than hypertension (P < 0.001), presence of dyspnea at hospital admission (P < 0.001), higher *Brixia* and CALL scores (P = 0.02 and 0.005, respectively), higher levels of gamma glutamyl transferase (P = 0.001), a blood sodium level > 145 mmol/L (P = 0.003), lymphocyte count < 1000 cells/µL (P = 0.03), higher levels of D-dimer (P = 0.004) and serum Interleukin-6 (P < 0.001), C reactive protein > 50 mg/L (P = 0.02), and corticosteroid therapy (P < 0.008).

**Table 2:** Univariate Cox analysis of risk factors for mortality of 48 patients with COVID-19 coming from a LTCF.

Variables	Non-survivors (n = 14)	Survivors (n = 34)	HR (95% IC)	P values
**Demographic information**				
Male sex	5 (35.7)	21 (61.7)	0.39 (0.13–1.19)	0.09
Age (years, mean ± SD)	85 ± 8	78 ± 13	1.04 (0.99–1.10)	0.09
**Comorbidities**				
Cancer	4 (28.6)	4 (11.7)	2.14 (0.67–6.85)	0.19
Cardiovascular diseases (CVD)	12 (85.7)	28 (82.3)	1.20 (0.27–5.37)	0.81
CVD other than hypertension	11 (78.8)	7 (20.6)	8.93 (2.47–32.30)	0.001
Psychiatric disorders	1 (7.1)	13 (38.2)	0.16 (0.02–1.22)	0.07
Neurologic diseases	9 (64.3)	17 (50)	1.61 (0.54–4.81)	0.39
Obesity	1 (7.1)	4 (11.7)	0.69 (0.09–5.29)	0.72
Diabetes mellitus	3 (21.4)	8 (23.5)	0.98 (0.27–3.52)	0.97
COPD	4 (28.6)	4 (11.7)	2.91 (0.91–9.32)	0.07
Chronic renal failure	5 (35.7)	14 (41.2)	0.86 (0.29–2.57)	0.78
> 2 comorbidities	7 (50)	18 (52.9)	0.95 (0.33–2.71)	0.92
**Symptoms**				
Days from symptoms to hospitalization (days, mean ± SD)	5 ± 3	6 ± 3	0.96 (0.78–1.18)	0.70
Fever	10 (71.4)	23 (67.6)	1.18 (0.37–3.77)	0.77
Cough	3 (21.4)	14 (41.2)	0.47 (0.13–1.68)	0.24
Dyspnea	5 (35.7)	0	13.77 (4.08–46.50)	< 0.001
Severe disease (NIH criteria)	8 (57.1)	16 (47.1)	1.40 (0.48–4.03)	0.53
**Polipharmacy**	11 (78.6)	25 (73.5	1.30 (0.36–4.66)	0.68
Number of medications (mean ± SD)	6 ± 3	6 ± 3	0.97 (0.82–1.15)	0.75
ACE/ARB	3 (21.4)	13 (38.2)	0.50 (0.14–1.81)	0.29
*Brixia* score (mean ± SD)	9 ± 2	7 ± 3	1.25 (1.03–1.50)	0.02
CALL core	12 ± 1	11 ± 1	2.02 (1.23–3.31)	0.005
Temperature ≥ 37.5 °C	3 (21.4)	4 (11.7)	2.13 (0.59–7.64)	0.24
Heart rate ≥ 85 beats per minute	6 (42.8)	10 (29.4)	1.74 (0.60–5.03)	0.30
Median blood pressure (mmHg, mean ± SD)	92 ± 17	93 ± 14	0.99 (0.96–1.03)	0.81
Oxygen saturation ≤ 94%	7 (50)	8 (23.5)	2.52 (0.88–7.20)	0.08
Glycemia (mg/dL, mean ± SD)	139 ± 72	121 ± 71	1.00 (0.99–1.00)	0.32
Creatinine ≥ 1.20 mg/dL	4 (28.6)	11 (32.3)	0.92 (0.29–2.93)	0.88
Aspartate transaminase (UI/L, mean ± SD)	132 ± 313	32 ± 14	1.00 (1.00–1.00)	0.23
Gamma glutamyl transferase (UI/L, mean ± SD)	99 ± 122	30 ± 22	1.00 (1.00–1.01)	< 0.001
Blood sodium level > 145 mmol/L	9 (64.3)	6 (17.6)	5.32 (1.77–16.01)	0.003
Ferritin level (ng/mL, mean ± SD)	732 ± 571	464 ± 336	1.00 (0.99–1.00)	0.06
Lactate dehydrogenase > 500 UI/L	10 (76.9)	19 (55.9)	2.18 (0.59–7.93)	0.23
Lymphocytes count < 1000 cells/µL	10 (71.4)	12 (35.3)	3.54 (1.11–11.30)	0.03
Platlets count (cells/µL, mean ± SD)	195 ± 88	197 ± 72	0.99 (0.99–1.00)	0.84
D-dimer (mg/L, mean ± SD)	5.41 ± 7.49	1.21 ± 1.05	1.11 (1.03–1.19)	0.004
C reactive protein > 50 mg/L	10 (71.4)	11 (32.2)	3.69 (1.15–11.79)	0.02
Interleukin-6 (pg/mL, mean ± SD)	125 ± 189	34 ± 22	1.005 (1.002–1.008)	< 0.001
Enoxaparin (therapeutic dose)	9 (64.3)	17 (50)	1.69 (0.57–5.05)	0.34
Hydroxychloroquine plus azytromicin therapy	9 (64.3)	28 (82.3)	0.49 (0.16–1.46)	0.20
Corticosteroid therapy	13 (92.8)	12 (35.3)	15.52 (2.02–118.98)	0.008

*CVD* cardiovascular diseases, *ACE-I* angiotensin-converting-enzyme inhibitors, *ARBs* angiotensin-receptor blockers, *NIH* National Institutes of Health.

At multivariable Cox regression, significant predictors of in-hospital mortality were: blood sodium level > 145 mmol/L (HR 9.12, 95% CI 2.15–38.52; P = 0.003), lymphocyte count < 1000 cells/µL (HR 7.45, 95% CI 1.81–30.68; P = 0.005), CVD other than hypertension (HR 6.41, 95% CI 1.51–27.22; P = 0.01), and higher IL-6 serum levels (pg/mL) (HR 1.005, 95% CI 1.001–1.009; P = 0.007) (Table 3).

**Table 3:** Cox regression analysis for in-hospital mortality in COVID-19 patients coming from a LTCF.

Variables	HR	(95% IC)	P values
Blood sodium level > 145 mmol/L	9.12	(2.15–38.52)	0.003
Lymphocytes count < 1000 cells/µL	7.45	(1.81–30.68)	0.005
CVD other than hypertension	6.41	(1.51–27.22)	0.01
Interleukin-6 blood level (pg/mL)	1.005	(1.001–1.009)	0.007

In terms of calibration, using the Grønnesby and Borgan test, the model displayed good calibration (P = 0.56).

By testing several values at increasing stepwise by 5, we identified 50 pg/mL as a cut-off. Consequently, we have performed a new Cox analysis including the variable IL-6 > 50 pg/mL which resulted to be independently associated with mortality (HR 4.50, 95% CI 1.28–15.84; P = 0.02) without changing the other results significantly (blood sodium level > 145 mmol/L [HR 8.55, 95% CI 1.93–37.71; P = 0.005], lymphocyte count < 1000 cells/µL [HR 11.08, 95% CI 2.42–50.70; P = 0.002], CVD other than hypertension [HR 8.58, 95% CI 2.01–36.72; P = 0.004] or the good calibration of the model (P = 0.90).

## Discussion

In the present study, we found that in-hospital mortality among elderly resident in a LTCF and diagnosed with COVID-19 was 32%. This finding is in line with (or slightly lower than) preliminary data estimated in LTCFs in Italy, where mortality has been reported as high as 37.4%, much lower than reported in other countries (49–64%)^8,12^. It has however to be recognised that we report herein the in-hospital mortality, not taking into account possible deaths occurred inside the LTCF. Notwithstanding this consideration, taking into account mean age of our patients (i.e., 80 years), in-hospital mortality rate in our cohort is in line with the case fatality rate (30.4%) among patients with age ranging from 80 and 89 years in the general population as reported by the Italian National Institutes of Health^13^. A significant percentage of our patients (84%) reported at least one symptom among fever, cough and/or dyspnea at hospital admission. In a cohort of residents of a LTCF during a COVID-19 outbreak in USA, only 13/23 (56%) of patients tested positive for SARS-CoV-2 presented clinical symptoms of COVID-19^14^. Also in two serial point-prevalence surveys conducted during a SARS-CoV-2 outbreak in a Skilled Nursing Facility, on a total of 48 residents who tested positive, 27 (56%) did not display symptoms at the time of the first survey but, of these, 24 subsequently developed symptoms so that the definitive rate of symptomatic patients was 93.7%^9^. Therefore, percentage of patients with symptoms in our study was in the range of what previously reported. However, comparisons among different studies are limited by types of symptoms (only fever, cough and/or dyspnea were considered in our cohort).

Multivariable Cox regression analysis showed that blood sodium level > 145 mmol/L, lymphocyte count < 1000 cells/µL, CVD other than hypertension, and higher IL-6 serum levels were independent predictors of in-hospital mortality.

In general, hypernatremia (defined as a sodium value higher than 145 mEq/L) is a condition frequently observed in patients (especially elderly) at the time of hospitalization and affects up to 9% of critical patients. Moreover, it has been reported that patients with hypernatremia have a significantly higher mortality rate compared to patients with normal values^15,16^. To the best of our knowledge, hypernatremia has been never recognized as independent risk factor for mortality in COVID-19 patients. Instead, of note, hypernatremia has been reported as a factor significantly associated with mortality in a small (n = 25) cohort of patients suffering from severe acute respiratory syndrome (SARS) and ARDS^17^. Hypernatremia has been identified as a potential surrogate marker of sepsis, especially in elderly because a severe systemic infection can lead to a significant free water deficit and subsequent hypernatremia^18^. In addition, some evidence also suggests that sodium could represent an important promoter of immune response, improving the function of macrophages and T-lymphocytes^19^. Although our finding should be confirmed by further studies, it could be assumed that hypernatremia may have a role as a marker related to the severity of the inflammatory state in COVID-19 elderly patients.

Lymphopenia and cardiovascular diseases have been widely described as factors associated with a worse outcome in cohorts of COVID-19 patients^3–6,20^. Our study confirms the important clinical impact of these factors also in elderly patients. Of note, in line other studies, in our patients there was not any difference for in-hospital mortality between patients who were receiving angiotensin-converting-enzyme (ACE) inhibitors or angiotensin-receptor blockers (ARBs) and those not treated with these drugs^3^.

The last independent predictor of mortality in our cohort was higher IL-6 serum levels. A cytokine storm, also known as cytokine release syndrome, has been suggested to be a major mechanism causing the more severe and often fatal clinical complications of COVID-19, such as ARDS and multi-organ dysfunction^21^. In particular, IL-6, which has been implicated in many immunological functions (e.g. B-cell stimulation and induction the production of acute phase proteins), can also activate the clotting pathway, vascular endothelial cells, and lead to a cytokine storm^22^. Gao et al. demonstrated that high levels of IL‐6 and D‐dimer were closely related to the occurrence of severe COVID‐19 in adult patients^23^. Moreover, Wan et al. demonstrated that IL-6 serum levels can predict transition from mild to severe infection^24^, and Liu et al. found that in Cox proportional hazard model IL-6 was an independent factor predicting severity of COVID-19 with a significant level > 32.1 pg/mL^25^. In line with these findings, in our cohort of COVID-19 elderly patients, higher IL-6 serum levels resulted to be independently associated with a higher risk of in-hospital mortality, suggesting its predictive value. Although it is difficult to identify a reliable cut-off which can predict the risk of death from our small cohort of patients, by testing several values at increasing stepwise by 5, we identified 50 pg/mL as a cut-off. Consequently, we have performed a new Cox analysis including the variable IL-6 > 50 pg/mL which resulted to be independently associated with mortality without changing the other results significantly.

The major limitation of this study is the small population size, which could not provide a clear degree of certainty to the presented results. It is, for example, the case of corticosteroid therapy, which apparently resulted to be associated with mortality at univariate analysis; indeed, we administered corticosteroids to patients with more severe diseases and this could represent a possible bias of the analysis. By contrast, the main strength is that patients were all infected in a limited period of time, so this is an incident cohort, and analysis of outcome predictors could be more reliable.

To the best of our knowledge, the present is the first study investigating clinical characteristics, mortality and prognostic factors of a complete cohort of patients during a SARS-CoV-2 outbreak in a LTCF. We found that in-hospital mortality was 32%, in line with fatality rates reported in elderly patients in Italy. Importantly, hypernatremia, lymphopenia, CVD other than hypertension, and higher IL-6 serum level appeared to be independent predictors of in-hospital mortality. Further larger studies are necessary to better understand and confirm our findings, in order to rapidly identify characteristics associated with an adverse outcome among elderly suffering from COVID-19 and provide a more aggressive monitoring and care.

## Materials and methods

We conducted a retrospective, single-centre, observational cohort study in order to describe clinical characteristics and baseline predictors of mortality of COVID-19 in hospitalized patients as part of an outbreak of SARS-CoV-2 infection in a LTCF.

Between March 27th, 2020 and May 6th, 2020, a total of 50 consecutive patients diagnosed with SARS-CoV-2 infection were transferred from a LTCF and admitted at Infectious and Tropical Disease Unit of “*Mater Domini*” Teaching Hospital, Catanzaro, Italy. The diagnosis of SARS-CoV-2 infection was established according to the WHO recommendations^26^.

This study was notified to the Ethics Committee of the Calabria Region on May 13th, 2020 and conducted in in accordance with the declaration of Helsinki. The study was carried out using retrospectively collected and anonymized data. In Italy, such studies do not require ethical approval by an Ethics Committee as determined by the Italian Drug Agency note 20 March 2008 (GU Serie Generale no. 76 31/3/2008). The need for written informed consent was waived for patients owing to the retrospective nature of the study.

### Data collection

Data collected from the hospital charts and the laboratory database included patient demographics, underlying diseases, previous drug therapies (including angiotensin-converting-enzyme [ACE] inhibitors and angiotensin-receptor blockers [ARBs]), reported COVID-19 symptoms (i.e., fever, cough, dyspnea), classification of COVID-19 disease according to National Institutes of Health (NIH) criteria^27^ clinical signs at hospital admission (i.e. body temperature, heart rate, median blood pressure, oxygen saturation level), laboratory findings at hospital admission (including serum interleukin-6 [IL-6], D-dimer, C reactive protein). All therapies administered for treatment of COVID-19 were also recorded. Chest X-ray (CXR) findings were classified according to the experimental CXR scoring system (*Brixia* score) validated by Borghesi et al. in hospitalized patients with COVID-19 pneumonia in order to quantify and monitor the severity and progression of COVID-19^28^. The CALL score was also calculated in order to predict the progression of COVID-19^29^.

All information were entered in case report forms and then recorded in a specific database. Two researchers independently reviewed the data collection forms to double check the collected data.

The outcome measured was in-hospital mortality during the study period. Survivor and non-survivor subgroups were compared in order to identify predictors of mortality.

### Statistical analysis

Univariate and multivariate analyses using the Cox proportional hazard regression model were conducted to identify independent risk factors for 21-day mortality. Values are expressed as means ± standard deviation (SD) (continuous variables) or as percentages of the group from which they were derived (categorical variables). Variables emerging from univariate analysis with P values of < 0.05 were included in the Cox regression model. Calibration (model fit) was assessed statistically using the Grønnesby and Borgan test. All statistical analyses were performed using the Intercooled Stata program, version 16, for Windows (Stata Corporation, College Station, Texas, USA). Description of statistical analysis was partially reported elsewhere^30^.
